# Manual of Human Microscopical Anatomy

**Published:** 1854-12

**Authors:** 


For sale by MILLER, ORTON, & MULLIGAN. H.

---

ART. X.—*Manual of Human Microscopical Anatomy.* By A. KÖLLIKER, Professor of Anatomy and Physiology in Wurzburg. Translated by GEO. BUSK, F. R. S., and THOMAS HUXLEY, F. R. S. Edited, with notes and additions, by J. DA COSTA, M. D. Philadelphia: Lippincott, Grambo & Co. 1854.

The literature of microscopical anatomy is becoming sufficiently full to offer chances for comparison. Few treatises on *Human* Microscopy, however, have as yet been republished in this country. Messrs. Blanchard & Lea have given us Todd and Bowman's Physiological Anatomy, but aside from this there was a great deficit in such works on this branch of knowledge as were easily accessible to the American anatomist. Gerber's General Anatomy, good, learned, accurate, but not comprehensive, has never been republished here; while Quekett's Lectures on Histology, are in the same category. These works, also, are rather general in their structure, elementary, or devoted to the description of type forms wherever found, whether in the vegetable or the animal kingdom. What we needed was a work on special histology, giving us an account of the various microscopic structure of different organs, going from one viscus or tissue to another in order, and reducing to a shapely convenience the mass of facts which have been accumulated by various observers.

Such a work is the one before us. Its style is interesting and attractive, and its arrangement admirable. Dr. Kölliker divides his book into two parts: the first devoted to general histology, in which the primitive tissues are described, commencing with the cell, and going on to the formation from it of epidermic, cartilaginous, elastic, connective, osseous, muscular, nervous, and glandular tissues. Then, having established his definitions, he takes up special histology, organ by organ; each receives a full consideration. The reader, in perusing it, will get rid of much mental confusion, and find his knowledge arranging itself in a definite and accessible form.

We are much indebted to the Sydenham Society for this translation, a labor which Dr. Da Costa had intended to perform himself until he found that he was anticipated by the London translators. In this dilemma Dr. Da Costa has sensibly adopted the English translation, adding to it such notes, or making such changes as were necessary.

The illustrations are excellent, and add much to the merit of the book, which we cordially commend to the medical public as the best of its kind.

